# Constriction Rings: The Missing Link

**Published:** 2007-12-28

**Authors:** Mark Kiehn, David Leshem, Ronald Zuker

**Affiliations:** Division of Plastic Surgery, University of Wisconsin Hospital and Clinics, Madison, Wisconsin; Department of Plastic & Reconstructive Surgery, Tel-Aviv Sourasky Medical Center, Tel-Aviv, Israel; Division of Plastic Surgery, Hospital for Sick Children, Toronto, Ontario, Canada

## Abstract

**Objective:** Amniotic band syndrome is an uncommon clinical entity with a spectrum of presentation including constriction rings, syndactyly, amputations, and spontaneous abortion. The bands themselves are not present at the time of birth. This has created controversy regarding the pathogenesis of the disorder. **Methods:** The unusual case of a neonate with a persistent band of fibrous amniotic material and the associated deformity of a constriction ring is evaluated. The significance of this unique case is discussed in the context of existing understanding of the syndrome. **Results:** The amniotic band was released in the neonatal intensive care unit shortly after birth. Despite removal of the band, the constriction ring persisted. **Conclusion:** The presence of a fibrous amniotic band at the site of a constriction ring is an extremely unusual finding. This case further demonstrates the importance of amniotic material in the etiology of constriction rings.

Amniotic band syndrome is an uncommon clinical entity that has been recognized for centuries.^1^,^2^ The manifestations of amniotic band syndrome range from constriction rings of the thorax, extremities, and head, to syndactyly,^1^,^3^–^5^ to amputations, and to spontaneous abortion.^1^ Seventy-seven percent of patients present with multiple anomalies.^6^ At the time of birth, effects of the process are noted, but causative constricting bands are generally not seen. The lack of direct evidence led to the development of 2 main theories of etiology. The intrinsic theory holds that germ cell deficiencies result in malformations of the affected parts.^4^,^7^,^8^ The extrinsic theory supports the role of bands of ruptured amnion in the creation of extrinsic compression that results in constriction rings and other deformities of the developing fetus.

In this report, the unusual case of neonate with postnatal persistence of an amniotic band and associated constriction is reported.

A female dichorionic, diamniotic twin born at the 27th week of gestation, was noted at birth to have a constriction ring at the distal aspect of her right leg (Fig 1). The constriction was circumferential, 3 mm in width, and involved the skin and subcutaneous tissues. A band of dark, inelastic fibrous material was present within the area of constriction. The right foot was well perfused with good color, warmth, and capillary refill, and there were no apparent deficits of motion or sensation. The foot and portion of the leg distal to the constriction were slightly edematous compared to the unaffected side.

The fibrous band was removed in the neonatal intensive care unit. The skin underlying the band was noted to be intact, and the band was easily removed (Fig 2). The tissue was sent to the department of pathology for evaluation. On histological evaluation, the specimen was found to be degenerated and no characterization beyond “fibrous tissue” was possible. The contour depression of the leg persisted following removal of the band (Fig 3). The foot and distal aspect of the leg continues to show signs of good perfusion and remains without evidence sensory or motor compromise (Fig 4). The edema of the foot and distal aspect of the leg were resolved despite the persistence of the constriction band.

The etiology of constriction rings has been a topic of debate since the recognition of the clinical entity. Some have speculated that the clinical findings result from malformations due to defective germ cell lines.^4^,^7^,^8^ This “intrinsic” theory attempted to account for the external findings and craniofacial, renal, cardiac, lung, and diaphragmatic anomalies.^10^ The “extrinsic” theory proposes that rupture of amnion and its separation from the chorion results in bands of tissue that are capable of entangling the developing fetus. Compression from these bands then results in deformity of the developing extremities, trunk, and head.^1^,^9^,^11^ Most cases are evaluated at the time of birth, when the deformities are noted, although a causative band is no longer present. Improved imaging modalities now allow for in utero diagnosis of these bands and determination of blood flow distal to the constriction.^12^,^13^ Some centers have performed intrauterine release of amniotic bands in an attempt to salvage limbs threatened with vascular insufficiency as a consequence of the constriction.^13^–^15^ The case presented in this report further demonstrates the importance of amniotic bands in the development of constriction rings and documents an unusual presentation of this condition. It is not clear what caused the persistence of the fibrous amniotic band in this case. It is possible that the patient's premature birth prevented complete degeneration of the band prior to birth. As is seen with most cases of constriction band syndrome, spontaneous resolution of the constriction did not occur despite removal of the fibrous band. Such improvement in contour generally requires release of the constriction and approximation of the soft tissues at the appropriate level.

## Figures and Tables

**Figure 1:**
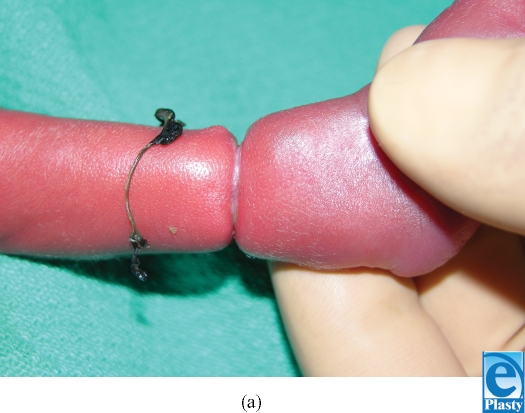
Constriction ring with persistent fibrous band at the distal aspect of the right leg.

**Figure 2:**
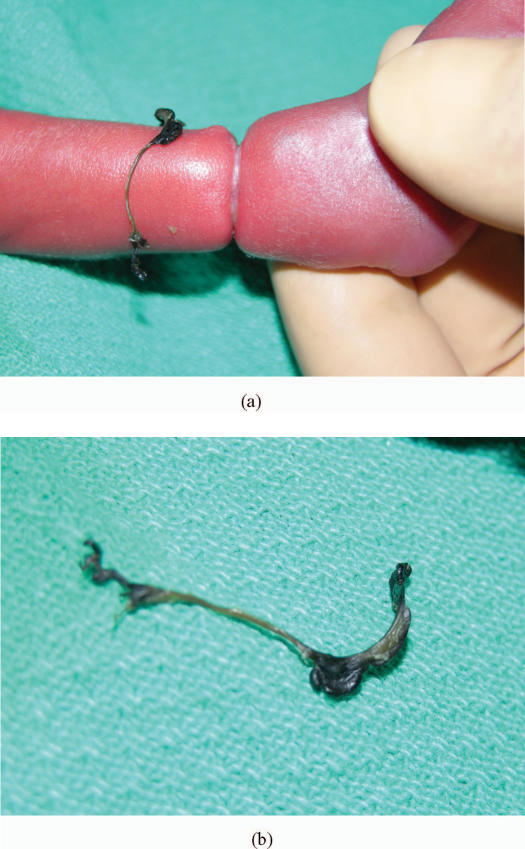
(a) Right leg after fibrous band is cut. (b) Persistent fibrous band.

**Figure 3:**
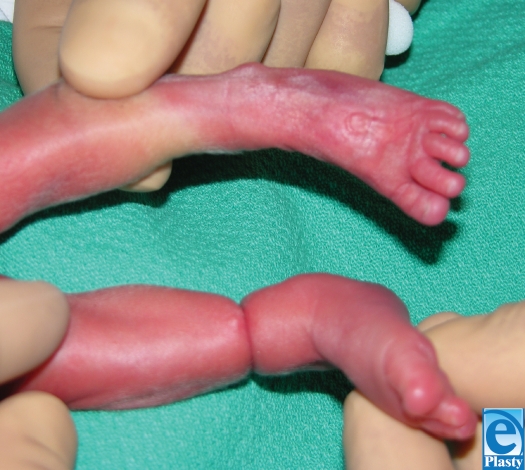
Comparison of affected and unaffected legs.

**Figure 4:**
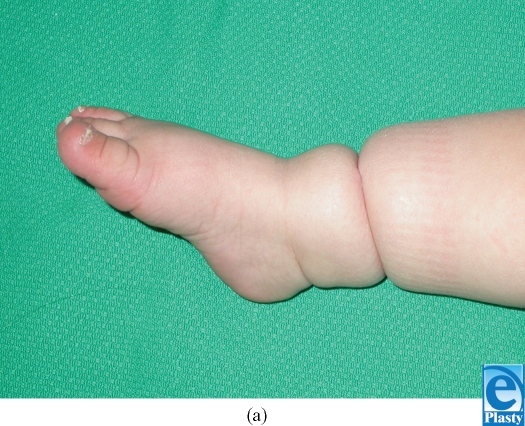
One-year following birth. (a) Plantar flexion and (b) dorsiflexion.

## References

[1] Higginbottom MC, Jones KL, Hall BD, Smith DW (1979). The amniotic band disruption complex: timing of amniotic rupture and variable spectra of consequent defects. J Pediatr.

[2] Light TR, Ogden JA (1993). Congenital constriction band syndrome. Pathophysiology and treatment. Yale J Biol Med.

[3] Askins G, Ger E (1988). Congenital constriction band syndrome. J Pediatr Orthop.

[4] Patterson TJ (1969). Syndactyly and ring constrictions. Proc R Soc Med.

[5] Yilmaz E, Dogan Y, Taskin E, Aygun D (2003). Amniotic band syndrome: congenital anular constrictions. J Eur Acad Dermatol Venereol.

[6] Baker CJ, Rudolph AJ (1971). Congenital ring constrictions and intrauterine amputations. Am J Dis Child.

[7] Streeter G (1930). Focal Deficiencies in fetal tissues and their relation to intrauterine amputations. Contrib Embryol Carnegie Inst.

[8] Moerman P, Fryns JP, Vandenberghe K, Lauweryns JM (1992). Constrictive amniotic bands, amniotic adhesions, and limb-body wall complex: discrete disruption sequences with pathogenetic overlap. Am J Med Genet.

[9] Torpin R (1965). Amniochorionic mesoblastic fibrous strings and amniotic bands: associated constricting fetal malformations or fetal death. Am J Obstet Gynecol.

[10] Bamforth JS (1992). Amniotic band sequence: streeter's hypothesis reexamined. Am J Med Genet.

[11] van der Meulen JC (1999). The amniotic band syndrome. Plast Reconstr Surg.

[12] Tadmor OP, Kreisberg GA, Achiron R, Porat S, Yagel S (1997). Limb amputation in amniotic band syndrome: serial ultrasonographic and Doppler observations. Ultrasound Obstet Gynecol.

[13] Ronderos-Dumit D, Briceno F, Navarro H, Sanchez N (2006). Endoscopic release of limb constriction rings in utero. Fetal Diagn Ther.

[14] Quintero RA, Morales WJ, Phillips J, Kalter CS, Angel JL (1997). In utero lysis of amniotic bands. Ultrasound Obstet Gynecol.

[15] Keswani SG, Johnson MP, Adzick NS (2003). In utero limb salvage: fetoscopic release of amniotic bands for threatened limb amputation. J Pediatr Surg.

